# Analysis of the history and spread of HIV-1 in Uganda using phylodynamics

**DOI:** 10.1099/vir.0.000107

**Published:** 2015-07

**Authors:** Gonzalo Yebra, Manon Ragonnet-Cronin, Deogratius Ssemwanga, Chris M. Parry, Christopher H. Logue, Patricia A. Cane, Pontiano Kaleebu, Andrew J. Leigh Brown

**Affiliations:** ^1^​ Institute of Evolutionary Biology, University of Edinburgh, Edinburgh, UK; ^2^​ MRC/UVRI, Uganda Research Unit on AIDS, Entebbe, Uganda; ^3^​ NADP Training, Public Health England, Porton, UK

## Abstract

HIV prevalence has decreased in Uganda since the 1990s, but remains substantial within high-risk groups. Here, we reconstruct the history and spread of HIV subtypes A1 and D in Uganda and explore the transmission dynamics in high-risk populations. We analysed HIV *pol* sequences from female sex workers in Kampala (*n* = 42), Lake Victoria fisher-folk (*n* = 46) and a rural clinical cohort (*n* = 74), together with publicly available sequences from adjacent regions in Uganda (*n* = 412) and newly generated sequences from samples taken in Kampala in 1986 (*n* = 12). Of the sequences from the three Ugandan populations, 60 (37.1 %) were classified as subtype D, 54 (33.3 %) as subtype A1, 31 (19.1 %) as A1/D recombinants, six (3.7 %) as subtype C, one (0.6 %) as subtype G and 10 (6.2 %) as other recombinants. Among the A1/D recombinants we identified a new candidate circulating recombinant form. Phylodynamic and phylogeographic analyses using BEAST indicated that the Ugandan epidemics originated in 1960 (1950–1968) for subtype A1 and 1973 (1970–1977) for D, in rural south-western Uganda with subsequent spread to Kampala. They also showed extensive interconnection with adjacent countries. The sequence analysis shows both epidemics grew exponentially during the 1970s–1980s and decreased from 1992, which agrees with HIV prevalence reports in Uganda. Inclusion of sequences from the 1980s indicated the origin of both epidemics was more recent than expected and substantially narrowed the confidence intervals in comparison to previous estimates. We identified three transmission clusters and ten pairs, none of them including patients from different populations, suggesting active transmission within a structured transmission network.

## Introduction

Human immunodeficiency virus type 1 (HIV-1) is one of the most devastating viral pathogens, particularly in sub-Saharan Africa (UNAIDS, 2013). HIV-1 group M (which is responsible for the pandemic) is subdivided into nine subtypes (A–D, F–H, J, K), and multiple inter-subtype recombinants (LANL, 2014a). Phylogenetic analysis of HIV genetic sequences (Volz *et al.*, 2013) allows the reconstruction of viral history and helps to understand the course of an epidemic over time (Hué *et al.*, 2005) and Bayesian statistical inference, integrating concepts of coalescent theory, is a powerful approach to reconstructing the spatial diffusion of the virus (Lemey *et al.*, 2009).

Phylodynamic studies have revealed that HIV-1 emerged in West Central Africa in the early 20th century (Keele *et al.*, 2006; Korber *et al.*, 2000; Worobey *et al.*, 2008) and diversified in the Congo River basin, where the highest viral diversity has been recorded (Vidal *et al.*, 2000). It is thought that HIV-1 subtypes A1 and D spread into East Africa after 1950 and disseminated exponentially during the 1970s, partially due to the interconnectivity between cities in the area (Gray *et al.*, 2009), although the timing of these events remains uncertain. In contrast, HIV spread south later, with a point prevalence as low as 0.1 % in retrospective testing of a large number of samples from Malawi in 1982, rising only to 2 % as late as 1988–9 (McCormack *et al.*, 2002). In Uganda, the first estimates of HIV prevalence revealed a rate of 11 % among pregnant women in Kampala in 1985 (Carswell & Lloyd, 1987), which rose to 30 % by the peak of the epidemic in 1992 (Stoneburner & Low-Beer, 2004) when 18 % of the general population of Uganda was infected. Introduction of educational and behavioural interventions on a national scale reduced the HIV burden (Green *et al.*, 2006; Uganda AIDS Commission, 2012) to a current adult prevalence of 7.2 % (UNAIDS, 2013). High-risk groups are now recognized to have much higher incidence than the general rural population. Among female sex workers in Kampala, incidence was 3.7/100 person years (Vandepitte *et al.*, 2011) and in the fishing communities of Lake Victoria it was recorded as 5/100 person years (Asiki *et al.*, 2011). In contrast, in the rural population of Masaka district incidence is now ten-fold lower than the high-risk populations, at 0.5/100 person years (Nsubuga *et al.*, 2013). While the high-risk populations could act as reservoirs for HIV infections in the general population, viral transmission dynamics between communities has not previously been investigated in sufficient detail to allow the probability of this spread to be estimated.

Here, we reconstruct the historical and spatial spread of HIV subtypes A1 and D within the Ugandan Lake Victoria basin (the most affected region in the country) using a substantial dataset of newly generated and existing sequences, which has not previously been analysed, and explore the transmission dynamics of the high-risk populations to quantify the level of intermixing among them.

## Results

### HIV subtype distribution in the study population

We analysed 162 *pol* sequences from three Ugandan populations: female sex workers (FSW) based in Kampala, adults from fishing communities of Lake Victoria (Fisher-Folk, ‘FF’) and patients from the rural clinical cohort (RCC) established in Masaka district (see Methods). Sixty of these sequences (37.1 %) were classified by SCUEAL as subtype D, 54 (33.3 %) as subtype A1, 31 (19.1 %) as A1/D recombinants, six (3.7 %) as subtype C, one (0.6 %) as subtype G and ten (6.2 %) as other recombinants. The last included three (1.8 %) complex recombinants, two C/D, two A/B (1.2 % each), one A/G, one A/C and one D/G (0.6 % each). In addition, 15 of the 54 (27.8 %) subtype A1 sequences were characterized by SCUEAL as intrasubtype recombinants, which were excluded from the evolutionary analyses (Table 1). The phylogenetic trees showed a star-like structure, compatible with a rapid increase in the HIV prevalence since the origin of the regional epidemic (Fig. S1, available in the online Supplementary Material). Among the A/D recombinants, we detected a phylogenetic clade of five sequences that shared similar breakpoints, whose details are explained below. We found no other lineage of phylogenetically close A/D recombinants with the same breakpoints.

**Table 1.** Distribution of HIV *pol* sequences in the study population according to subtype and cohort

	Cohort (%)			Total
HIV-1 variant	FSW	Lake Victoria FF	Masaka RCC
Subtype A1	19 (45.2)	16 (34.8)	19 (25.7)	54
Subtype D	8 (19.0)	18 (39.1)	34 (45.9)	60
Other subtypes	5 (11.9)	1 (2.2)	1 (1.3)	7
Inter-subtype recombinants	10 (23.8)	11 (21.7)	20 (27.0)	41
**All**	**42**	**46**	**74**	**162**

There was significant diversity in subtype prevalence among the three populations (χ^2^_2_ = 8.55; *P*<0.02), with the largest difference being that between the Kampala-based FSW group (45.1 % subtype A1, 19 % subtype D) and the RCC (25.7 % subtype A1, 45.9 % subtype D; χ^2^_1_ = 7.22; *P*<0.003). In the Lake Victoria FF, the proportion of subtypes D (39.1 %) and A1 (34.8 %) was intermediate. The fraction of inter-subtype recombinants was very similar in FSW (23.8 %), RCC (27.0 %) and FF (23.9 %).

### Analysis of phylogenetic clusters and pairs

**Fig. 1.**
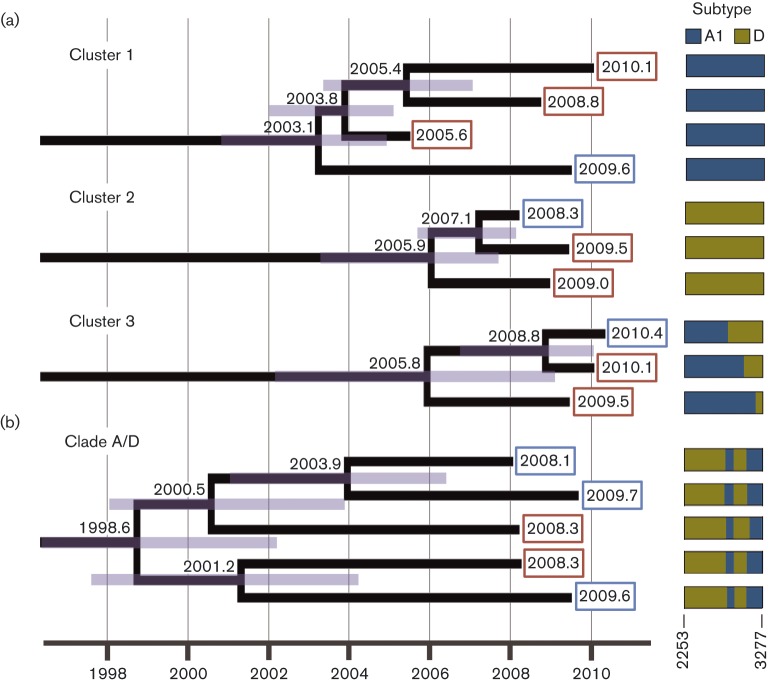
(a) Three clusters identified among 162 HIV-infected individuals. (b) Complete A/D recombinant lineage identified through possession of common breakpoints. Clusters were defined using a maximum pairwise genetic distance of 1.5 %. Subtype assignations are indicated in the panel on the right (between position 2253 to 3277; HXB2). All clusters and the recombinant clade correspond to sequences from the RCC in Masaka District. The numbers at the nodes indicate the MRCA and the purple rectangles indicate the 95 % highest posterior density. The numbers at the tips represent the sampling time for each sequence, and the colour of the squares denotes the patient’s sex (blue, male; red, female). The horizontal axis is expressed in calendar years.

Three clusters (≥3 sequences) and ten pairs were identified in the three Ugandan populations with a maximum pairwise genetic distance less than 1.5 %. These involved 30 of the 162 (18.5 %) sequences (Table 2): 20 from RCC and 10 from FF. They corresponded to subtype A1 (one cluster of four sequences – cluster 1 – and two pairs), D (one cluster of three sequences – cluster 2 – and four pairs) and A/D recombinants (one cluster of three sequences – cluster 3 – and four pairs) (Fig. 1a).

**Table 2.** Distribution of phylogenetic networks according to size and viral variant

HIV-1 variant	Cluster size			
2	3	4	All
Subtype A1	2	0	1	3
Subtype D	4	1	0	5
A1/D recombinants	4	1	0	5
**All**	**10**	**2**	**1**	**13**

The largest cluster (cluster 1, which included four subtype A1-infected subjects sampled between 2005 and 2010) comprised three women and one man linked through past sexual relationships. Cluster 3, the triplet infected by an A/D recombinant (sampled in 2009–2010) comprised a heterosexual couple and a woman who reported a past sexual partner in common with the woman in the couple. These three A/D recombinant sequences presented different breakpoints, at positions 2818, 3022 and 3178 (HXB2 reference coordinates) respectively. Cluster 3 was characterized on the basis of the 565 nt segment of A1 sequence they all had in common (Fig. 1a). Cluster 2 (involving subtype D sequences sampled in 2008–09) also included a heterosexual couple and a second woman, this time with no known epidemiological linkage to the couple. The estimated most recent common ancestor (MRCA) of these three clusters (Fig. 1a) was 2003.1 for cluster 1 (A1), 2006.0 for cluster 2 (D) and 2005.8 for cluster 3 (A1/D), respectively. All three clusters corresponded exclusively to patients from the RCC. Of the ten pairs, five included only RCC patients and five only FF subjects. Seven out of the ten pairs included a man and a woman (three of which were confirmed sexual couples), and three included two women (with one pair connected by a known non-sampled male partner). The average MRCA of the pairs was 2006.5. All the clusters and pairs were confirmed in the Bayesian tree, and supported by a posterior probability of 1.

We calculated ‘cluster depth’ as the difference between the MRCA and the date of the most recent sample within the cluster. This depth was 5 years on average (range: 3.6–7.0) for the clusters and 3 years (range: 0.3–7.4) for the pairs.

A quarter (20, 27.0 %) of the 74 RCC sequences were included in clusters or pairs. This proportion was slightly (but non-significantly) lower for FF sequences (10, 21.7 %), which were more interspersed in the trees (Fig. S1). No sequences from FSW were included in clusters and no clusters or pairs included sequences from more than one population.

### A1/D recombinant lineage

A clade of five A/D recombinant sequences with very similar breakpoints was discovered among RCC patients (three male, two female) sampled between 2008 and 2009 (Fig. 1b). These sequences all showed a multiple D/A1/D/A1 recombination pattern with three breakpoints in each sequence at positions ~2813, 2920 and 3083 (HXB2 reference coordinates), distinct from any published circulating recombinant forms (CRFs) and any of the remaining A/D sequences found here. The individuals, aged 16–33 years at sampling, were not recorded as being sexual partners. The MRCA of this group was estimated to be 1998.6 (95 % highest posterior density, HPD, 1994.8–2002.3).

### Phylodynamics of subtypes A1 and D

We selected those *pol* sequences that belonged to pure subtypes A1 (*n* = 208) and D (*n* = 273) and applied Bayesian MCMC inference to these datasets (Table 3). For both subtypes, the GMRF skyride demographic model provided the best fit to the data and it yielded evolutionary rate estimates of 1.3×10^−3^ (9.1×10^−4^–1.7×10^−3^) substitutions/site year^−1^ for A1 and 1.9×10^−3^ (1.5–2.3×10^−3^) for subtype D in the initial analysis based on recent sequences alone. The corresponding MRCAs were 1945 (1923–1963) and 1966 (1958–1972). However, when the sequences from 1986 were added (three A1 and eight D), the evolutionary rate estimates rose to 1.7×10^−3^ (1.4–2.1×10^−3^) and 2.4×10^−3^ (2.1–2.7×10^−3^) substitutions/site year^−1^ for A1 and D, respectively. The MRCAs for each were brought forward to 1960 (1950–1968) for A1 and 1973 (1970–1977) for D. To confirm this observation, we constructed maximum-likelihood (ML) trees with RAxML and PhyML corresponding to both the A1 and D datasets, with and without the sequences from 1986. We used two additional independent methods for assigning dates to trees, Path-O-Gen (http://tree.bio.ed.ac.uk/software/pathogen/) and least square dating (LSD, http://www.atgc-montpellier.fr/LSD/), to estimate the TMRCA and we observed the TMRCAs were more recent in 15/16 analyses (data not shown).

**Table 3.** Distribution of HIV *pol* sequences used for the phylodynamic and phylogeographic analyses according to subtype and location

	HIV-1 variant		
Location and cohort	Subtype A1	Subtype D	Total
**Kampala**	**125**	**87**	**212**
FSW	13	7	20
LANLdb	109	72	181
‘Old’ sequences (1986)	3	8	11
**Entebbe (LANLdb)**	**20**	**13**	**33**
**Lake Victoria (FF)**	**10**	**16**	**26**
**Rural south-west**	**53**	**157**	**210**
Masaka district (RCC)	12	30	42
Rakai district (LANLdb)	41	127	168
**All**	**208**	**273**	**481**

LANLdb, Los Alamos National Laboratory HIV database.

From the original datasets, sequences identified as intra-subtype recombinants by SCUEAL (*n* = 15, all subtype A1) and sequences with particularly high evolutionary rates according to Path-O-Gen (four subtype A1 and seven subtype D) were excluded to improve the evolutionary analysis in BEAST (see Methods).

To assess the difference in evolutionary rates between subtypes, we performed a reciprocal test. The rate for subtype D was estimated with a fixed MRCA corresponding to that estimated for subtype A1 (1959.6) and vice versa for the evolutionary rate of subtype A1 with the MRCA of subtype D (1973.4). As expected, this restriction caused different evolutionary rates [now 2.4×10^−3^ (2.2–2.6×10^3^) for subtype A1 and 1.7×10^−3^ (1.5–1.8×10^−3^) for subtype D]. We compared the fit to the data for the constrained versus unconstrained models using Bayes factors (BFs), and in both cases we observed that the unconstrained models for both subtypes A1 and D fit the data substantially better than the inappropriately constrained models (log_10_ BF, 11.9 and 13.6, respectively). We conclude that the difference between the TMRCAs is real.

**Fig. 2.**
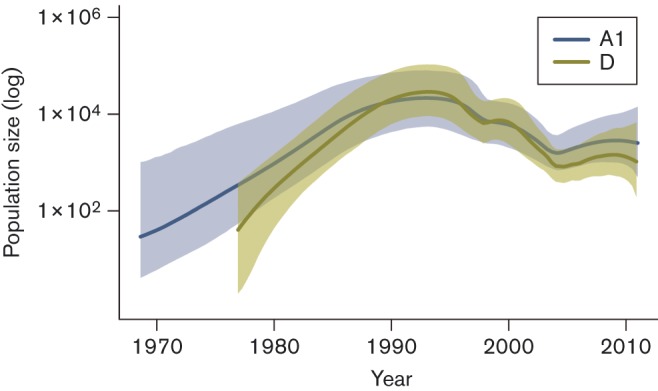
Bayesian Skyride plot showing the changes in the estimated viral effective population size for subtypes A1 and D in Uganda across time. The horizontal axis is expressed in calendar years.

The inclusion of sequences sampled in 1986 also improved the convergence of the MCMC chains, producing higher values of estimated sample size (ESS) and reducing the 95 % HPD of the parameters sampled. The Bayesian Skyride plots (Fig. 2) show the same temporal trends in viral population growth for both subtypes, with approximately exponential growth in the 1970s and 1980s reaching a maximum in the early 1990s (when the population size of subtype D exceeded that of subtype A1). Subsequently, a decrease in estimated effective population number for both subtypes occurred until a final period of stabilization (or moderate increase) after the mid-2000s.

### Phylogeography of subtypes A1 and D

**Fig. 3.**
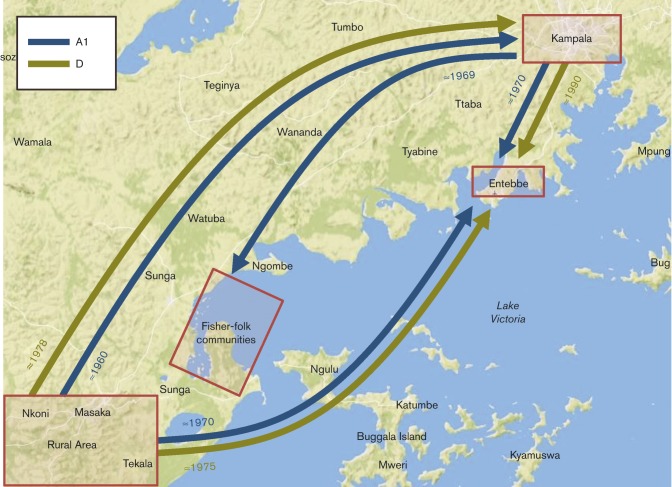
Inferred routes of HIV subtypes A1 (in blue) and D (in green) spread between the four Ugandan locations considered, highlighted with red rectangles. Only the statistically significant routes (supported by BF >3) are shown. The dates accompanying each arrow indicate approximately when these movements occurred in time. This figure was constructed using maps from MapBox (www.mapbox.com).

A phylogeographic analysis using the discrete traits approach implemented in BEAST (Lemey *et al.*, 2009) was performed using four locations: Kampala, Entebbe, the shores of Lake Victoria and the rural south-west region (Table 3). For both subtypes, the root of the tree was located in the rural south-western region (posterior probability = 1). In subtype A1, the diffusion analysis showed a strongly supported initial movement to Kampala (BF >35) with subsequent spread from Kampala and the south-west to both Entebbe (BF >8) and Lake Victoria (BF >5) (Fig. 3).

For subtype D, the analysis showed a similar pattern but with independent initial introductions from the rural south-west, to Kampala (BF >6), Entebbe (BF >4) and Lake Victoria (in that order) and a later movement from Kampala to Entebbe (BF >4).

Extending the analysis to other East African countries in the ML trees did not reveal any clear separation by country for either subtype. Instead, all sequences were interspersed in the trees with only a few country-specific clusters (data not shown).

A phylogeographic analysis, which included the most similar non-Ugandan sequences, suggested multiple migration events between the Ugandan locations and different cities of Tanzania, Kenya and, for subtype A1, Rwanda (Fig. S2). However, these movements were more weakly supported than those described within Uganda.

## Discussion

We have reconstructed the history and spread of the predominant HIV-1 subtypes in the Ugandan Lake Victoria basin using a new dataset, which included sequences from high-risk populations (FSW and FF), an RCC, sequences from a publicly available database and 11 newly generated sequences from samples taken in 1986 in Kampala. Our phylodynamic analysis revealed that the subtype A1 epidemic in the country originated around 1960, while that of subtype D initiated in the early 1970s. Gray and colleagues (Gray *et al.*, 2009) also identified an earlier introduction of subtype A, but had substantially older dates of introduction for each, of 1948 for subtype A1 and the early 1960s for subtype D. In both subtypes, the virus spread from south-western Uganda eastwards to Kampala. HIV-1 sequences from rural areas were more likely to be found in clusters than sequences from higher risk groups and we found no evidence of intermixing among these communities. Although the sample size and the sampling coverage were low, this could suggest an epidemic compartmentalized by risk group.

### HIV variants circulating in Uganda

Our results agree with larger datasets that had previously revealed (Ssemwanga *et al.*, 2012b) the predominance of subtype A1 in the Ugandan urban areas and that of subtype D in rural, south-western Uganda, with only a few cases of other subtypes (C, G). However, we also observed in all populations large numbers of recombinants (30 % of the initial dataset) involving the two main subtypes, which indicates a high degree of intermixing between these variants. Among the rural cohort from Masaka district we discovered a clade of five A/D recombinant sequences with very similar recombination patterns in the *pol* gene, distinct from the two currently described CRFs involving A1 and D in *pol* (CRF35_AD and CRF50_AD; LANL, 2014a) and from the other A/D recombinants found. Further study, including full-length genome data, will be needed to establish whether this constitutes a new CRF. Given the increasing presence of recombinants in the pandemic, either unique or as CRFs (especially in regions where different subtypes co-circulate; LANL, 2014b), more effort should be made to characterize the contribution that such recombinants make to the epidemic, as they are usually excluded from phylogenetic analyses.

### HIV transmission clusters in Uganda

Over 25 % of sequences from the rural Masaka cohort study (RCC) were linked in pairs or larger clusters at a 1.5 % genetic distance criterion. A slightly smaller proportion of sequences from FF was linked (22 %) but only in pairs, and no links were observed that involved FSW. The detection of clustering in a relatively small sample of the RCC could indicate that in such an area partner choice may be restricted geographically. A recent study (Grabowski *et al.*, 2014) conducted in rural communities in the Rakai district combined phylogenetic analysis with interview data. One conclusion reached was that extra-household infections were more likely also to be extra-community. This conclusion was based on interview data as was not possible to distinguish within-community from between-community infections on the basis of phylogenetic analysis of the data available. However, samples from high-risk communities were not included in that analysis, which may have reduced their chances of detecting links between communities, and the lower availability of sequences from these high-risk communities is a significant limitation of this study also.

In Uganda, FSW and the FF communities represent the main high-risk groups with an HIV prevalence of 37 % and 29 %, respectively (Asiki *et al.*, 2011; Vandepitte *et al.*, 2011), while HIV prevalence is lower in rural farming communities (around 7 % in 2009) (Uganda AIDS Commission, 2012). Previous work has revealed a considerable sexual partner mixing within Ugandan fishing communities (Nazziwa *et al.*, 2013) and FSW (and their clients) in Kampala (Ssemwanga *et al.*, 2012b), but epidemiological (Pickering *et al.*, 1997) and phylogenetic (Yirrell *et al.*, 1998) studies have observed little mixing between residents of the rural areas and Lake Victoria fishing villages, a result we confirm here as we found no cases of inter-population interaction.

### Introduction and spread of subtypes A1 and D in Uganda

Our estimates of the emergence of the HIV-1 epidemic in Uganda of 1960 (1950–1968) for subtype A1 and 1973 (1970–1977) for subtype D are substantially more recent than previously thought (Gray *et al.*, 2009). The tight and non-overlapping confidence intervals suggest a significantly older introduction of subtype A1 in Uganda. The estimates were substantially improved by the inclusion of sequences from 1986, which allowed substantial improvements in the estimate of evolutionary rates through better calibration of the molecular clock.

The evolutionary rates estimated for subtypes A1 (1.7×10^−3^ substitutions/site year^−1^) and D (2.4×10^−3^) in Uganda lie in the usual range described for HIV-1 *pol* sequences [1–3×10^−3^ substitutions/site year^−1^ (Abecasis *et al.*, 2009; Hué *et al.*, 2005; Maljkovic Berry *et al.*, 2007; Wertheim *et al.*, 2012)] but differ significantly from each other. A higher evolutionary rate for subtype D could be associated with the faster disease progression and CD4^+^ T-cell decline observed in subtype D when compared to A1 (Baeten *et al.*, 2007; Kaleebu *et al.*, 2002; Kiwanuka *et al.*, 2008). It has been suggested that subtype D is less transmissible than A1 (Kiwanuka *et al.*, 2009), and that there is a declining relative prevalence in the Rakai district (1994–2002) (Conroy *et al.*, 2010), but this has not been confirmed in the adjacent Masaka district in a similar period (1990–2000) (Yirrell *et al.*, 2004). Demographic and/or historical events during the exponential growth of both subtypes in the 1980s could have had a major effect on dissemination of these viruses.

### The rural south-west as the epicentre of the Ugandan epidemic

Although the limited statistical support prevents us from definite conclusions, our results pointed to the south-western rural areas as the geographical origin of the Ugandan epidemic. For the subtype D dataset it might appear that this result could be influenced by the higher number of sequences from these areas, but we obtained the same result for A1, for which sequences from Kampala were the majority (Table 1). This hypothesis agrees with the fact that, despite the higher HIV prevalence in urban areas, the first AIDS cases in Uganda were reported in the Rakai district, in the rural south-west (Serwadda *et al.*, 1985). The extreme south-west of Uganda borders the Democratic Republic of Congo (DRC), where HIV-1 subtype M originated and is crossed by the trans-African highway on its way to Masaka and Kampala; the importance of this transport artery in HIV transmission has been well documented (Gray *et al.*, 2009). Nevertheless, it should be borne in mind that conclusions from all phylogeographic analyses are strongly dependent on the origin of the samples included, which is a major limitation. Although we have used all *pol* sequences currently available, large areas of Uganda remain underrepresented and despite subsampling, some remain overrepresented. A more extended and structured sampling strategy, although difficult to achieve in practice, would be needed in order to remove this bias.

### Conclusions

We reconstructed the history of HIV in the most affected region in Uganda, a country with more than an estimated 1.5 million infected people. Subtypes A1 and D, which currently dominate the epidemic in the region, entered Uganda through its south-west, with subtype D entering almost 15 years later than subtype A1. Both spread exponentially during the 1970s and 1980s and showed extensive viral migration towards and from the HIV epidemics of surrounding countries and high levels of recombination. In addition, although the sample size was small and the sampling coverage low, we evaluated the HIV transmission dynamics in three different populations, but found little mixing between them, which might suggest a relatively compartmentalized epidemic.

## Methods

### 

#### Study population.

We analysed 162 HIV partial *pol* sequences (mean length = 1,071 bp) sampled between 2005 and 2010. They came from three cohorts in Uganda: 42 sequences from FSW based in Kampala (Vandepitte *et al.*, 2011); 46 sequences from adults from fishing communities of Lake Victoria (Fisher-Folk, ‘FF’) (Nazziwa *et al.*, 2013); and 74 from the RCC established in Masaka district at south-west Uganda (Ssemwanga *et al.*, 2012a).

In order to improve the evolutionary analyses (see below), we included 11 *pol* sequences (eight subtype D, three subtype A1) newly amplified from samples taken in 1986 during a serological survey in Mulago and Rubaga hospitals in Kampala (Carswell, 1987). These samples were shipped to the Centre for Applied Microbiology at Porton Down (now Public Health England, UK), tested using an HIV ELISA test and stored at −80 °C since then. The sequencing procedure, for which new ethical permission was obtained, was previously described (Cane, 2011).

#### HIV subtyping and clustering.

The sequences from the three Ugandan cohorts were classified using SCUEAL (Kosakovsky Pond *et al.*, 2009). Phylogenetic HIV transmission clusters (containing >two sequences) and pairs were identified in ML trees constructed with RAxML (Stamatakis *et al.*, 2008) with a maximum pairwise genetic distance within the cluster of 1.5 % using the ClusterPicker v1.3 (Ragonnet-Cronin *et al.*, 2013). This parameter is the best approximation of time to MRCA in time-stamped phylogenies (Leigh Brown *et al.*, 2011). Anonymized epidemiological information linked to the sequence data (concerning sex, age, sexual partners, etc.) was included in the analysis in order to compare these records with the reconstructed clusters or pairs.

To estimate the MRCAs of clusters and pairs, we applied Bayesian MCMC inference to these 162 sequences using BEAST v1.7.5 (Drummond *et al.*, 2012). We used an uncorrelated log-normal relaxed molecular clock with the SRD06 model of nucleotide substitution (Shapiro *et al.*, 2006), and tested several demographic models (constant size, exponential growth, logistic growth, Bayesian Skyline and GMRF Skyride) whose results were compared by means of the BFs using Tracer v1.5 (http://tree.bio.ed.ac.uk/software/tracer/). The dataset ran for 100 million generations sampling evolutionary parameters every 10 000th generation. The tree samples were used to generate a maximum clade credibility (MCC) tree after removing a 10 % burn-in using TreeAnnotator v1.7.5 (http://beast.bio.ed.ac.uk/TreeAnnotator). A Bayesian Skyride plot was generated using Tracer v1.5 to represent the changes over time of the viral effective population size (Minin *et al.*, 2008).

#### Evolutionary analyses of HIV subtypes A1 and D in Uganda.

All *pol* sequences from the Los Alamos National Laboratory (LANL) Database (LANL, 2014b) of subtypes A1 (*n* = 176) and D (*n* = 235) from Uganda with sampling city information (mainly Kampala, Entebbe and Rakai district) and date (1992–2005) were included. The datasets were analysed with Path-O-Gen v1.4 (tree.bio.ed.ac.uk/software/pathogen) to identify sequences with particularly high evolutionary rates (*n* = 20), which were discarded to improve the analysis. Including the sequences from 1986, the final datasets used for the subsequent analyses comprised 208 A1 and 273 D sequences (see Table 1 for a breakdown of sequences by location and subtype).

We applied Bayesian MCMC inference with the same conditions described above, processing two independent runs of 200 million generations that were combined afterwards. 

Finally, we conducted a Bayesian phylogeographic analysis using an asymmetrical discrete traits analysis implemented in BEAST v1.7.5 to identify migration pathways of the virus between the different locations in Uganda. Bayesian stochastic search variable selection (BSSVS) was employed to reconstruct the geographical locations of the ancestral states (Lemey *et al.*, 2009) contained in a subsample of the posterior tree distribution of the BEAST runs.

The migration routes indicated by the MCC tree (i.e. the changes of state in the nodes) were visualized using Google Earth (http://earth.google.com) and SPREAD (http://www.kuleuven.be/aidslab/phylogeography/SPREAD.html). SPREAD was also used to identify statistically well-supported migration routes (those with BF >3).

A second phylogeographic analysis was performed to investigate HIV transmission between the Ugandan locations and neighbouring countries in Eastern Africa. We downloaded all unique subtype A1 (*n* = 1,505) and D (*n* = 307) sequences in the LANL Database from Tanzania, Kenya, Rwanda, DRC and Sudan. We constructed initial trees using FastTree v2 (Price *et al.*, 2010) and included the ten closest sequences to each sequence in our dataset in a second phylogeographic analysis (using ViroBLAST; Deng *et al.*, 2007). These reference sequences (sampled between 1992 and 2011) were from Kenya (55 subtype A1 and 50 subtype D; from Nairobi, Mombasa, Kilifi, Kisumu and Kericho), Tanzania (33 A1 and 41 D; from Mbeya, Moshi, Mwanza and Bukoba), Rwanda (21 A1 and two D), Sudan (four D; from Khartoum) and DRC (two A1; from Kinshasa). The phylogeographic analysis was performed as above.
